# Treatment with Intravenous Methylprednisolone in Patients with Graves’ Orbitopathy Significantly Affects Adrenal Function: Assessment of Serum, Salivary Cortisol and Serum Dehydroepiandrosterone Sulfate

**DOI:** 10.3390/jcm9103233

**Published:** 2020-10-09

**Authors:** Katarzyna Pelewicz, Sebastian Szewczyk, Piotr Miśkiewicz

**Affiliations:** 1 Department of Internal Medicine and Endocrinology, Medical University of Warsaw, 02–091 Warsaw, Poland; katarzyna.pelewicz@gmail.com; 2 Student’s Scientific Circle “Endocrinus”, Department of Internal Medicine and Endocrinology, Medical University of Warsaw, 02–091 Warsaw, Poland; s.szewczyk098@gmail.com

**Keywords:** adrenal insufficiency, dehydroepiandrosterone sulfate, glucocorticoids, Graves’ ophthalmopathy, Graves’ orbitopathy, methylprednisolone, salivary cortisol, serum cortisol

## Abstract

Treatment of active, moderate-to-severe Graves’ orbitopathy (GO) is the administration of intravenous methylprednisolone (IVMP). IVMP may be followed by additional therapy with oral prednisone. The aim of this study was to analyze the impact of IVMP on adrenal function by evaluation of serum, salivary cortisol and serum dehydroepiandrosterone sulfate (DHEA-S). Fourteen patients received IVMP treatment (cumulative dose of 4.5 g in 12 weekly infusions) followed by oral prednisone (for three months). All patients showed normal adrenal function before the 12th IVMP pulse and one patient was diagnosed with secondary adrenal insufficiency (AI) after prednisone treatment. DHEA-S was significantly lower before the 12th IVMP pulse and after oral prednisone (*p* = 0.015 and *p* = 0.00002, respectively) in comparison to evaluation before therapy. DHEA-S levels were below the reference range in one and three patients before the 12th IVMP pulse and after prednisone therapy, respectively. We observed decreased serum (*p* = 0.05) and salivary (*p* = 0.011) cortisol levels after oral prednisone therapy in comparison to evaluation before therapy. Treatment with IVMP in a cumulative dose of 4.5 g affects adrenal function, causing more severe impairment of DHEA-S secretion than that of cortisol but does not cause secondary AI. Additional therapy with oral glucocorticoids after IVMP can cause secondary AI.

## 1. Introduction

Graves’ orbitopathy (GO) is an extra-thyroidal manifestation of Graves’ disease. It presents as a group of ocular symptoms caused by an enlargement of extraocular muscles and orbital adipose tissue due to autoimmunological inflammation. According to the European Group on Graves’ Orbitopathy (EUGOGO) protocol, management of active, moderate-to-severe GO is the administration of intravenous methylprednisolone (IVMP) pulses [1]. Treatment with glucocorticoids (GCs) may cause suppression of the hypothalamic–pituitary–adrenal (HPA) axis and precipitate adrenal insufficiency (AI). The influence of intravenous GCs on the HPA axis has not been sufficiently investigated.

The aim of this study was to analyze possible adverse effects of therapy with IVMP on adrenal function and to assess the use of salivary cortisol and serum dehydroepiandrosterone sulfate (DHEA-S) to evaluate the HPA axis function after treatment with IVMP and oral prednisone in patients with GO.

## 2. Material and Methods

### 2.1. Patients

Fourteen individuals (11 females) with active, moderate-to-severe GO according to the EUGOGO criteria treated with IVMP and prednisone, were included in the study. The presence of ≥1 of the following features of GO: inconstant or constant diplopia, lid retraction ≥ 2 mm, moderate or severe soft-tissue involvement or exophthalmos ≥ 23 mm was categorized as moderate-to-severe GO [2]. Patients were admitted to the Department of Internal Medicine and Endocrinology, Medical University of Warsaw, between 2013 and 2016. The inclusion criterion was euthyroidism within the last three months before the study. Exclusion criteria were diagnosis of AI, treatment with GCs or medication altering the plasma cortisol binding globulin (CBG) and serum DHEA-S levels within the last six months before the study and medical conditions altering CBG levels (e.g., hepatitis, cirrhosis, diabetes, nephrotic syndrome, polycystic ovary syndrome, hypoproteinemia and inflammation). The study was approved by the Commission of Bioethics at the Medical University of Warsaw, Poland (KB 42/2011; 15 March 2011) and conducted with the written consent of all participants, in accordance with the Declaration of Helsinki.

### 2.2. Study Design and Treatment

Four factors were statistically analyzed: serum cortisol, salivary cortisol, serum DHEA-S and plasma adrenocorticotropic hormone (ACTH) levels. The factors were assessed at three time points: directly before administration of the 1st and 12th IVMP pulses and after the cessation of oral prednisone therapy. Blood and saliva samples were obtained between 8:00 and 9:00 a.m. For saliva collection, a Salivette^®^ commercial device (Sarstedt, Nümbrecht, Germany) was used. Patients were advised to obtain saliva samples after fasting and without brushing their teeth. Serum and saliva samples were stored at −20 °C until analysis.

We administered a cumulative dose of 4.5 g of IVMP every week following the protocol (6 weekly infusions of 0.5 g and afterward, 6 weekly infusions of 0.25 g) according to EUGOGO recommendations (1). Following this, patients received 30 mg of oral prednisone in a single daily dose, tapered on a two-week schedule to 5 mg/day over 3 months. Afterward, oral substitution of hydrocortisone 10 mg/day was administered until the day of the examination of morning serum and salivary cortisol, DHEA-S and ACTH (at least seven days after prednisone cessation). Secondary AI was ruled out in patients who did not present clinical features of AI (weakness, fatigue, orthostatic hypotension, muscle pain, hyperpigmentation, nausea, weight loss, hypoglycemia) and had morning serum cortisol level > 10 μg/dL. In patients with morning serum cortisol < 10 μg/dL high-dose (250 µg), a synthetic ACTH stimulation test was performed. Serum cortisol was evaluated before, 30 min and 60 min after the injection. Secondary AI was ruled out if the serum cortisol level was >18 μg/dL after stimulation with synthetic ACTH, and the patient showed a lack of symptoms of AI. In these patients, oral hydrocortisone was discontinued. In patients with a diagnosis of secondary AI, we continued therapy with hydrocortisone 10 mg/day. A synthetic ACTH stimulation test was then performed every three months until a proper response in the ACTH stimulation test was observed. At that point, we discontinued hydrocortisone supplementation.

### 2.3. Assays

Serum and salivary cortisol concentrations were measured using a first-generation Elecsys^®^ cortisol assay (Roche Diagnostics Limited, Burgess Hill, UK). Serum DHEA-S and plasma ACTH levels were measured using Elecsys^®^ DHEA-S assay (Roche Diagnostics GmbH, Mannheim, Germany) and Elecsys^®^ ACTH assay (Roche Diagnostics Limited, Burgess Hill, UK), respectively. ACTH stimulation tests were performed with synthetic ACTH (0.25 mg Synacthen^®^ Novartis, Basel, Switzerland). The normal ranges of morning salivary cortisol were established at 0.11–1.29 ug/dL, based on healthy control group values between the 2.5th and 97.5th percentile. For serum DHEA-S concentration, we used age- and sex-adjusted reference ranges.

### 2.4. Statistical Analysis

Statistical analyses were performed using the STATISTICA 13.3.721.1 64-bit (PL) program. The results are expressed as mean ± standard deviation. The distribution of variables was measured using the Shapiro–Wilk test. A parametric test was used for the data with normal distribution—in this case, the paired *t*-test. If the result from the Shapiro–Wilk test was statistically significant (*p* ≤ 0.05), the Wilcoxon test was used to compare the variables’ nonparametric data. The decrease or increase of a given parameter was compared with the Student’s *t*-test and the Wilcoxon test. The results were considered statistically significant with *p*-value ≤ 0.05.

## 3. Results

At the beginning of the treatment, all patients presented with DHEA-S levels within age and sex-specific reference intervals. Before the 12th IVMP pulse, one of 14 (7%) patients had a DHEA-S level below the reference range. After oral prednisone therapy, three of 14 (21%) patients presented with DHEA-S levels below the reference range; one of these patients also had a morning serum cortisol level < 10 µg/dL.

Morning serum cortisol levels < 10 µg/dL were observed in three (21%) patients before the 12th IVMP pulse and in six (43%) patients after oral prednisone therapy. They underwent evaluation with a high-dose (250 µg) synthetic ACTH stimulation test. All three patients with decreased morning serum cortisol levels (<10 µg/dL) before the 12th IVMP pulse showed a proper response (serum cortisol level > 18 µg/dL) in the ACTH stimulation test. After oral prednisone treatment, five out of six patients with decreased morning serum cortisol levels showed a proper response in the ACTH stimulation test. One patient was diagnosed with secondary AI (serum cortisol in ACTH stimulation tests were 0′: 5.53 μg/dL, 30′: 14.61 μg/dL; 60′: 17.37 μg/dL).

**Figure 1:**
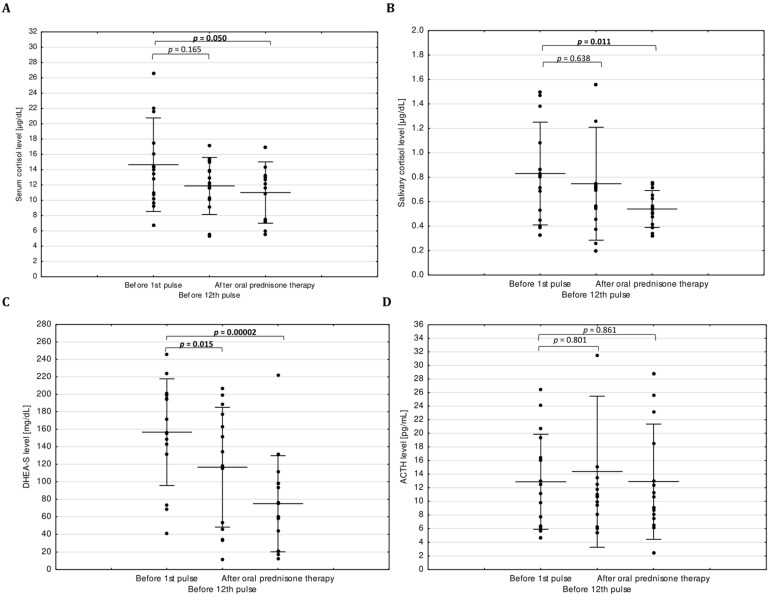
Comparison of variables during intravenous methylprednisolone treatment. (**A**) and (**B**) show cortisol levels in serum and saliva, respectively; (**C**) shows serum DHEA-S levels; (**D**) shows plasma ACTH levels. Abbreviations: ACTH, adrenocorticotropic hormone; DHEA-S, dehydroepiandrosterone sulfate; normal ranges: serum cortisol 6.2–19.4 µg/dL; salivary cortisol 0.11–1.29 µg/dL; ACTH 7.2–63.6 pg/mL; serum DHEA-S age and sex-adjusted reference ranges were used.

We found a statistically significant decrease of serum DHEA-S and no statistically significant decrease of serum cortisol and salivary cortisol (*p* = 0.015, *p* = 0.165 and *p* = 0.638, respectively) before administration of 12th IVMP pulse in comparison to evaluation before treatment. All patients fulfilled the criteria (laboratory and clinical) of normal adrenal function (Table 1; Figure 1).

After oral prednisone therapy, we observed statistically significant decreases of serum DHEA-S, serum and salivary cortisol (*p* = 0.00002, *p* = 0.05 and *p* = 0.011, respectively) in comparison to assessment before treatment.

The difference between ACTH levels at three time points during the treatment was not statistically significant. No statistically significant correlation between the age of the patients and serum DHEA-S, serum and salivary cortisol level changes was observed. The results are presented in Table 1 and Figure 1.

## 4. Discussion

Exogenous GCs may have a negative influence on the HPA axis, as secondary AI is frequently reported after cessation of GCs treatment. Although IVMP has been proven to cause various side effects [3], its impact on adrenal function has not been clearly established. Nonetheless, IVMP is widely used in many disorders, including inflammatory, autoimmune diseases. Determination of secondary AI remains challenging as diagnostic procedures are still being improved. Evaluation of morning serum cortisol levels or peak serum cortisol levels 30 and 60 min after stimulation with synthetic corticotropin is the recommended confirmatory test for AI [4]. Serum cortisol is bound to CBG and albumin in 90% and 10% of cases, respectively [5,6]. The states that reduce (inflammation and/or rare genetic syndromes) or increase (estrogen intake, pregnancy) these protein levels need to be considered in the interpretation of serum cortisol levels [7]. Salivary cortisol reflects the free fraction of cortisol, as it does not depend on albumin and CBG levels. Collecting a sample of salivary cortisol is noninvasive and may be done at home. For this reason, salivary cortisol shows potential as a more valuable screening biomarker of HPA axis dysfunction [8,9]. Dehydroepiandrosterone and DHEA-S are adrenal androgens also used in the assessment of the HPA axis. In clinical diagnostics, DHEA-S is used more frequently because of its longer half-life and lack of circulating diurnal variations [10].

In our research, we did not find a statistically significant decrease of serum and salivary cortisol before administration of the 12th IVMP pulse in comparison to evaluation before treatment, while one study showed a statistically significant decrease of serum cortisol concentration before administration of the 12th IVMP pulse [11]. The results of our study show that none of the patients developed secondary AI at the cessation of IVMP treatment in a cumulative dose of 4.5 g. Previous studies did not prove secondary AI after therapy with standard EUGOGO protocol (cumulative dose of 4.5 g IVMP) as well [11,12,13]. However, dynamic testing with synthetic ACTH has low sensitivity in ruling out secondary AI, especially when considering short-lasting (less than 4–6 weeks) AI [14]. For this reason, we evaluated the impact of IVMP on adrenal function using for the first-time salivary cortisol and DHEA-S level.

Although we did not observe secondary AI at the cessation of IVMP treatment, our study points to a statistically significant decrease of DHEA-S levels between the first and last pulse of IVMP. These results lead to the conclusion that therapy with IVMP in a cumulative dose of 4.5 g may significantly affect the HPA axis, as it leads to a more severe decrease of DHEA-S secretion than that of cortisol; therefore, therapy with a higher cumulative dose may possibly lead to secondary AI. Assessment of adrenal function should be considered after cessation of treatment with intravenous GCs, especially in patients receiving higher single and cumulative doses of IVMP. Measuring serum DHEA-S concentration may be helpful in assessing adrenal function before and after IVMP therapy of GO. Al-Aridi, Abdelmannan et al. [15] recommend measurements of baseline serum cortisol and serum DHEA-S levels in establishing the diagnosis of AI. They indicate that only very low (≤5 μg/dL) or clearly elevated levels of baseline serum cortisol measurements are helpful, whereas a normal age- and sex-adjusted serum DHEA-S level practically rules out the diagnosis of AI. Charoensri et al. [16] observed that normal age- and gender-specific DHEA-S levels predict intact HPA axis function with a sensitivity of 87.1%, a specificity of 86.7% and a positive predictive value of 93.1%.

After three further months of therapy with oral prednisone, we found one case of secondary AI and observed a statistically significant decrease of both serum DHEA-S and serum and salivary cortisol in comparison to evaluation before treatment. These results show that the use of oral GCs even in a relatively short period with low, tapered doses after IVMP administration in GO treatment may lead to secondary AI. Evaluation of the HPA axis after additional oral GCs treatment should be mandatory.

The use of salivary cortisol in the diagnosis of AI has been investigated in a great deal of research. In two independent studies [17,18], it has been shown that morning salivary cortisol is sensitive enough to be an alternative method for the ACTH stimulation test in the diagnosis of AI. The application of salivary cortisol measurement is limited by deficiently standardized assays and the lack of a single, validated reference range, as it was primarily intended for serum cortisol analysis [19]. Roche does not provide the lower cutoff point for the first-generation assay for morning salivary cortisol. In our study, we used the lower cutoff point established by the laboratory of the Department of Endocrinology (0.11 μg/dL). Deutschbein et al. estimated a similar lower cutoff for salivary cortisol level of 3.2 nmol/L (0.12 μg/dL with conversion factor for nmol/L to μg/dL: divide by 27.59) when screening for secondary AI [20]. Another study showed yet different cutoffs for the first generation assay being 6.14 nmol/L at waking and 5.42 nmol/L one-hour post-waking [21]. In our study, measurements of salivary cortisol levels did not prove to be useful in diagnosing secondary AI. After oral prednisone treatment, we did not find a decreased morning salivary cortisol concentration in the patient who was simultaneously diagnosed with AI using the synthetic ACTH stimulation test. According to recent studies [21,22], the second-generation assay shows a better correlation with liquid chromatography–tandem mass spectrometry (LC–MS/MS), the gold standard for cortisol measurement, compared to Cortisol I assay. Further research on the Roche Cortisol II assay may determine the use of salivary cortisol in the diagnostic of AI in patients with GO treated with IVMP and oral prednisone.

Regarding ACTH levels, the morning changes of ACTH are associated with pulsatile secretion and its very short half-life. In our study, the median levels of ACTH oscillated around 13 pg/mL, which is rather low. This could be an explanation for the fact that the ACTH levels did not change simultaneously with the decrease of DHEA-S and cortisol levels.

The present study has possible limitations, the main being small groups of patients and limited follow-up.

## 5. Conclusions

Treatment of active, moderate-to-severe GO with IVMP in a cumulative dose of 4.5 g causes a statistically significant decrease of DHEA-s levels. Therapy with IVMP in a cumulative dose of 4.5 g affects adrenal function, causing more severe impairment of DHEA-S secretion than that of cortisol but does not lead to secondary AI. Treatment with oral GCs after IVMP pulses, even for a relatively short period of time with low, tapered doses, can lead to secondary AI. Evaluation of the HPA axis after additional oral GCs treatment should be mandatory.

Further research with a greater group of patients treated according to schedule with higher doses of IVPM (6 weekly infusions of 0.75 g followed by 6 weekly infusions of 0.5 g) is needed to evaluate possible changes in serum, salivary cortisol and serum DHEA-S levels.

## Figures and Tables

**Table 1:** The results at three time points.

Variables	Before 1st Pulse	Before 12th Pulse	After Oral Prednisone Therapy
TSC (µg/dL)	14.46 ± 5.72	11.93 ± 3.56	10.90 ± 3.98 ^a^
SC (µg/dL)	0.82 ± 0.40	0.73 ± 0.43	0.54 ± 0.14 ^b^
DHEA-S (µg/dL)	155.60 ± 66.17	115.2 ± 74.18 ^a^	76.1 ± 58.97 ^c^
ACTH (pg/mL)	12.99 ± 7.48	13.49 ± 10.04	9.1 ± 8.72

Data are expressed as mean with standard deviation. Abbreviations: TSC, total serum cortisol (normal range: 6.2–19.4 µg/dL); SC, salivary cortisol (normal range: 0.11–1.29 µg/dL); DHEA-S, dehydroepiandrosterone sulfate (serum DHEA-S age- and sex-adjusted reference ranges were used); ACTH, adrenocorticotropic hormone (normal range: 7.2–63.6 pg/mL); statistically significant results are bolded; ^a^ *p* ≤ 0.05; ^b^ *p* ≤ 0.01; ^c^ *p* ≤ 0.00005, *p* values refer to comparisons to measurements before the 1st pulse.

## References

[1] Bartalena L., Baldeschi L., Boboridis K., Eckstein A., Kahaly G.J., Marcocci C., Perros P., Salvi M., Wiersinga W.M. (2016). The 2016 European Thyroid Association/European Group on Graves’ Orbitopathy Guidelines for the Management of Graves’ Orbitopathy. Eur. Thyroid J..

[2] Bartalena L., Baldeschi L., Dickinson A., Eckstein A., Kendall-Taylor P., Marcocci C., Mourits M., Perros P., Boboridis K., Boschi A. (2008). Consensus statement of the European Group on Graves’ orbitopathy (EUGOGO) on management of GO. Eur. J. Endocrinol..

[3] Miśkiewicz P., Kryczka A., Ambroziak U., Rutkowska B., Główczyńska R., Opolski G., Kahaly G., Bednarczuk T. (2014). Is high dose intravenous methylprednisolone pulse therapy in patients with Graves’ orbitopathy safe?. Endokrynol. Pol..

[4] Bornstein S.R., Allolio B., Arlt W., Barthel A., Don-wauchope A., Hammer G.D., Husebye E.S., Merke D.P., Murad H., Stratakis C.A. (2016). Diagnosis and Treatment of Primary Adrenal Insufficiency: An Endocrine Society Clinical Practice Guideline. J. Clin. Endocrinol. Metab..

[5] Mendel C.M., Kuhn R.W., Weisiger R.A., Cavalieri R.R., Siiteri P.K., Cunha G.R., Murai J.T. (1989). Uptake of cortisol by the perfused rat liver: Validity of the free hormone hypothesis applied to cortisol. Endocrinology.

[6] Mendel C.M., Miller M.B., Siiteri P.K., Murai J.T. (1990). Rates of dissociation of steroid and thyroid hormones from human serum albumin. J. Steroid Biochem. Mol. Biol..

[7] Nenke M.A., Zeng A., Meyer E.J., Lewis J.G., Rankin W., Johnston J., Kireta S., Jesudason S., Torpy D.J. (2017). Differential Effects of Estrogen on Corticosteroid-Binding Globulin Forms Suggests Reduced Cleavage in Pregnancy. J. Endocr. Soc..

[8] Ceccato F., Marcelli G., Martino M., Concettoni C., Brugia M., Trementino L., Michetti G., Arnaldi G. (2019). The diagnostic accuracy of increased late night salivary cortisol for Cushing’s syndrome: A real-life prospective study. J. Endocrinol. Invest..

[9] Langelaan M.L.P., Kisters J.M.H., Oosterwerff M.M., Boer A.K. (2018). Salivary cortisol in the diagnosis of adrenal insufficiency: Cost efficient and patient friendly. Endocr. Connect..

[10] Haning R.V., Hackett R.J., Boothroid R.I., Canick J.A. (1990). Steroid sulphatase activity in the human ovarian corpus luteum, stroma, and follicle: Comparison to activity in other tissues and the placenta. J. Steroid Biochem..

[11] Ambroziak U., Bluszcz G., Bednarczuk T., Miśkiewicz P. (2017). The influence of Graves’ orbitopathy treatment with intravenous glucocorticoids on adrenal function. Endokrynol. Pol..

[12] Jespersen S., Nygaard B., Kristensen L.Ø. (2015). Methylprednisolone Pulse Treatment of Graves’ Ophthalmopathy is Not Associated with Secondary Adrenocortical Insufficiency. Eur. Thyroid J..

[13] Giotaki Z., Fountas A., Tsirouki T., Bargiota A., Tigas S., Tsatsoulis A. (2015). Adrenal reserve following treatment of graves’ orbitopathy with intravenous glucocorticoids. Thyroid.

[14] Ospina N.S., Al Nofal A., Bancos I., Javed A., Benkhadra K., Kapoor E., Lteif A.N., Natt N., Murad H. (2016). ACTH Stimulation Tests for the Diagnosis of Adrenal Insufficiency: Systematic Review and Meta-Analysis. J. Clin. Endocrinol. Metab..

[15] Al-Aridi R., Abdelmannan D., Arafah B.M. (2011). Biochemical diagnosis of adrenal insufficiency: The added value of dehydroepiandrosterone sulfate measurements. Endocr. Pract..

[16] Charoensri S., Chailurkit L., Muntham D., Bunnag P. (2017). Serum dehydroepiandrosterone sulfate in assessing the integrity of the hypothalamic-pituitary-adrenal axis. J. Clin. Transl. Endocrinol..

[17] Patel R.S., Shaw S.R., McIntyre H.E., McGarry G.W., Wallace A.M. (2004). Morning salivary cortisol versus short Synacthen test as a test of adrenal suppression. Ann. Clin. Biochem..

[18] Ulhaq I., Ahmad T., Khoja A., Islam N. (2019). Morning cortisol as an alternative to short synecthan test for the diagnosis of primary adrenal insufficiency. Pak. J. Med. Sci..

[19] Bae Y.J., Gaudl A., Jaeger S., Stadelmann S., Hiemisch A., Kiess W., Willenberg A., Schaab M., Von Klitzing K., Thiery J. (2016). Immunoassay or LC-MS/MS for the measurement of salivary cortisol in children?. Clin. Chem. Lab. Med..

[20] Deutschbein T., Broecker-Preuss M., Flitsch J., Jaeger A., Althoff R., Walz M.K., Mann K., Petersenn S. (2012). Salivary cortisol as a diagnostic tool for Cushing’s syndrome and adrenal insufficiency: Improved screening by an automatic immunoassay. Eur. J. Endocrinol..

[21] Gagnon N., Fréchette I., Mallet P.L., Dubé J., Houde G., Fink G.D. (2018). Establishment of reference intervals for the salivary cortisol circadian cycle, by electrochemiluminescence (ECLIA), in healthy adults. Clin. Biochem..

[22] Vogeser M., Kratzsch J., Bae Y.J., Bruegel M., Ceglarek U., Fiers T., Gaudl A., Kurka H., Milczynski C., Prat Knoll C. (2017). Multicenter performance evaluation of a second generation cortisol assay. Clin. Chem. Lab. Med..

